# Indian Ocean Crossroads: Human Genetic Origin and Population Structure in the Maldives

**DOI:** 10.1002/ajpa.22256

**Published:** 2013-03-21

**Authors:** Jeroen Pijpe, Alex Voogt, Mannis Oven, Peter Henneman, Kristiaan J Gaag, Manfred Kayser, Peter Knijff

**Affiliations:** 1 Department of Human Genetics, Leiden University Medical CenterPostzone S5, 2300 RC Leiden, The Netherlands; 2 Division of Anthropology, American Museum of Natural HistoryCentral Park West at 79th Street, New York, NY, 10024, USA; 3 Department of Forensic Molecular Biology, Erasmus MC University Medical Center Rotterdam3000, CA Rotterdam, The Netherlands

**Keywords:** Y chromosome, mitochondrial DNA, migration, Indo-Aryan languages, South Asia

## Abstract

The Maldives are an 850 km-long string of atolls located centrally in the northern Indian Ocean basin. Because of this geographic situation, the present-day Maldivian population has potential for uncovering genetic signatures of historic migration events in the region. We therefore studied autosomal DNA-, mitochondrial DNA-, and Y-chromosomal DNA markers in a representative sample of 141 unrelated Maldivians, with 119 from six major settlements. We found a total of 63 different mtDNA haplotypes that could be allocated to 29 mtDNA haplogroups, mostly within the M, R, and U clades. We found 66 different Y-STR haplotypes in 10 Y-chromosome haplogroups, predominantly H1, J2, L, R1a1a, and R2. Parental admixture analysis for mtDNA- and Y-haplogroup data indicates a strong genetic link between the Maldive Islands and mainland South Asia, and excludes significant gene flow from Southeast Asia. Paternal admixture from West Asia is detected, but cannot be distinguished from admixture from South Asia. Maternal admixture from West Asia is excluded. Within the Maldives, we find a subtle genetic substructure in all marker systems that is not directly related to geographic distance or linguistic dialect. We found reduced Y-STR diversity and reduced male-mediated gene flow between atolls, suggesting independent male founder effects for each atoll. Detected reduced female-mediated gene flow between atolls confirms a Maldives-specific history of matrilocality. In conclusion, our new genetic data agree with the commonly reported Maldivian ancestry in South Asia, but furthermore suggest multiple, independent immigration events and asymmetrical migration of females and males across the archipelago. Am J Phys Anthropol 151:58–67, 2013. © 2013 Wiley Periodicals, Inc.

The Indian Ocean has been an important corridor for human migrations, and it has been travelled since the classical era (Hourani, 1995). Northern coastal trading route networks emerged around 2000 BP (years Before Present), when civilizations in East Africa; West, South, and East Asia; and the Mediterranean coasts expanded and developed their trading routes (Hogendorn and Johnson, 1986; Hourani, 1995). This Indian Ocean trade network was further developed during the Arab conquest starting around 1200 BP, during contact with China around the same time, and expanded into a global trade network with the arrival of Europeans after 500 BP (Chauduri, 1985). The interaction of such a variety of people during this time has provided opportunities for cultural and genetic admixture. Recent studies highlight unique admixture of Southeast Asians, South Asians, and Africans (from Central and East Africa) in the Malagasy of Madagascar and on the Comoros islands (Hurles et al., 2005; Ruivo et al., 2009; Tofanelli et al., 2009; Cox et al., 2012). The process of migration to Madagascar is still poorly understood. In a study that simulates seafaring routes across the Indian Ocean to explain the settling of Madagascar from Southeast Asia, the Maldives emerge in the center of such seafaring, and suggest that the Maldives could have been an important stopping point for voyages to Madagascar (Fitzpatrick and Callaghan, 2008).

The Maldives consist of an 850-km long string of atolls situated north to south some 400 km off the Kerala coast of the Indian subcontinent. Compared to other countries in the region, data on the origins of the Maldivians are scarce. Archaeological evidence indicates that human migration and settlement in the Maldives goes back at least 2,000 years (Bell, 1940; Fritz, 2002). The Maldives were the main producer of cowrie shells (*Cypraea* sp.) that were used as a currency throughout the classical old world and even reached West Africa (Hogendorn and Johnson, 1986). Language studies and historical records point to a historical relationship of the Maldivian language, Dhivehi, with the Indo-Aryan language of Sri Lanka, Sinhalese, making it the southernmost Indo-European language at the time the Maldives were populated (Fritz, 2002). Present Maldivian cultural and religious identity is strongly influenced by 800 years of Islamic culture (Bell, 1940; Maloney, 1980; Taj al-Din, 1982). Before Islam, Buddhism was the major religion, thought to be brought with Sinhala settlers from Sri Lanka (Maloney, 1980; Vitharana, 1997). Popular Maldivian board and card games suggest links with Southeast Asia (de Voogt, 2000) and South Asia (de Voogt, 2009).

Patterns of historical migration and gene flow within the Maldives are largely unknown. The long distances within the Maldives promote a relative isolation of islands, atolls and atoll groups. Its history includes female rulers—Sultanas—and a matrilineal tradition that may have changed over time to a patrilineal and patrilocal system under the influence of Islam (Metcalf, 2009). Noticeable differences between female and male gene flow can be expected between islands, if the present-day population is a mix of descendants from the original matrilocal people and descendants from more recent immigrants with a patrilocal tradition. The northern islands may have experienced a different migration and settlement pattern compared to the southern islands that are up to 800 km away. Only the three southernmost atolls (Gaafu, Gnaviyani, and Addoo) conducted, on their own, commercial activities with Sri Lanka until recently (Bell, 1940). The Maldivian subdialects are divided into two main groups: a northern and a southern dialect. The latter is again restricted to the three southernmost atolls (Fritz, 2002). Finally, it is noted that inhabitants of some islands claim a partly different ancestry than the widely acknowledged South Indian origin. Some islanders of Feridhoo claim to partly descend from African immigrants, which might explain the African influence that is still found in the islands' musical tradition (L. Reurich, personal communication).

It thus seems that the cultural and linguistic data convey a complex history of migration and settlement into the area that might be difficult to disentangle. This uncertainty about Maldivian history remains without additional reliable historical data. In contrast, a study of the vertically transmitted genetic variation in the present-day population may significantly advance our understanding of the ethnic origins and diversity of the Maldivian population. The population genetic variation in the Maldives is not independent from cultural transmission since successful migrants may confound cultural and genetic variation and remove most of the aboriginal genes and culture, as can be seen in Madagascar (Hurles et al., 2005; Tofanelli et al., 2009). Because of their relative isolation, islands are particularly prone to such processes. However, despite their dependence on cultural processes, genetic analyses are able to detect remnants of historic population processes. In addition, genetic research is particularly powerful in distinguishing gene flow between males and females. In migration events, sex-biased gene flow is the rule rather than the exception for most populations (Seielstad et al., 1998; Destro-Bisol et al., 2004; Heckel et al., 2005; De Filippo et al., 2011), and we expect this to be true in the Maldives as well. Thus far, no studies have explored the genetic ancestry of Maldivians, with the exception of a 1998 study limited to beta-thalassemia genes in beta-thalassemic patients (Furuumi et al., 1998) that concluded that the observed variation was best explained by a multi-ethnic origin from regions across the Indian Ocean, in particular from West Asia.

The aim of this study is twofold. First, we investigate the parental female- and male-mediated gene flow and admixture from neighboring regions across the Indian Ocean to the Maldives to identify the most likely regions of origin of the Maldivians. Second, we assess whether the historical, anthropological, and linguistic factors described above confirm the population genetic diversity and structure within the Maldives. Hereto, we studied autosomal DNA-, mitochondrial DNA-, and Y-chromosomal DNA markers in a representative sample of 141 unrelated Maldivians from six major settlements that are residing up to 800 km apart.

This study is timely. The predicted rising sea levels due to global warming may have the catastrophic consequence of submergence of the low-lying Maldives and hence the forced displacement of its people (IPCC, 2001), although recent studies have contested this particular danger to the Maldives (Morner et al., 2004). Strikingly, the island of Kandholhudhoo (in Raa atoll) that is included in our sample had to be deserted in the aftermath of the 2004 Indian Ocean tsunami.

## MATERIALS AND METHODS

### Population samples

**Fig. 1:**
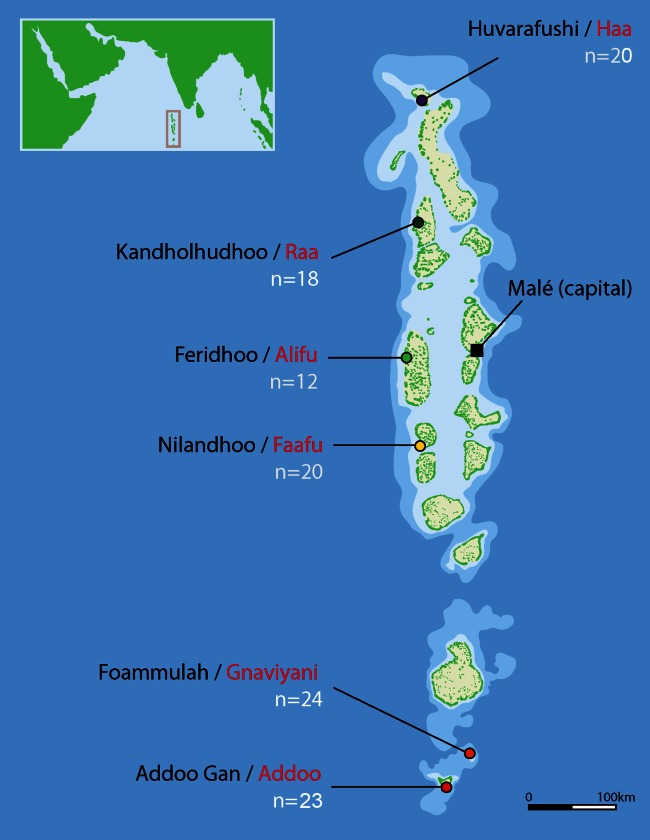
The Maldives geography and their location in the Indian Ocean (insert top-left). The six main sample locations are indicated by colored dots. The island names are followed by the atoll names, and the sample sizes are indicated.

In 2003, 141 mouth swab samples were collected at six medical clinics including Malé Hospital, the only large medical facility in the Maldives. Unrelated individuals, primarily from six main settlements across the Maldives that reported both parents to originate from the same island, were requested to participate. See Figure 1 for sampling locations and Table 1 for population details. DNA was isolated from buccal swabs using spin columns, as described by the manufacturer (Qiagen). Informed consent was obtained from all participants, and approval for the procedure was given by the Maldivian government, using the guidelines of the Leiden University Medical Center ethical committee.

**TABLE 1:** Maldives population details used in this study

								Sample size				Genetic data		
									
Island name	Atoll name	Atoll codein figures	Dialect	Population size^a^ Island/Atoll		Latitude^b^	Longitude^b^	Females	Males	Total	Autosomal STRs	mtDNA	Y-SNPs	Y-STRs
Huvarafushi	Haa Alifu	Haa	Northern	2,252	13,733	6.98N	72.89E	1	19	20	19	20	19	19
Kandholhudhoo	Raa	Raa	Northern	3,157	15,331	5.62N	72.86E	3	17	20	17	20	17	17
Feridhoo	Alifu	Ali	Northern	551	4,995	4.05N	72.73E	2	10	12	10	12	10	10
Nilandhoo	Faafu	Faa	Northern	1,268	3,864	3.06N	72.89E		20	20	20	20	20	20
Foammulah	Gnaviyani	Gna	Southern	7,645	7,645	0.30S	73.42E		24	24	24	24	24	24
Addoo Gan	Addoo	Add	Southern	2,883	17,980	0.70S	73.16E	3	20	23	20	23	20	20
Other	Male, Baa, Noonu, Haa Alifu, Gaafu Dhaalu, Gaafu Alifu, Laamu			Total population Maldives 186950		-	-	6	16	22	-	22	16	-
Total								15	126	141	110	141	126	110

a 2004 Census.

b UTM coordinates.

### Genetic analyses

#### mtDNA genotyping

We genotyped 17 mtDNA coding-region single nucleotide polymorphisms (SNPs) using a multiplex single-base primer extension (SNaPshot, Life Technologies) assay as described in Quintans et al. (2004). In addition, we sequenced hypervariable segments 1 (HVS1; nucleotide position (np) 16,001–16,410) and 2 (HVS2; np 51–401) within the mtDNA control region by Sanger sequencing of PCR amplified fragments on an ABI 3100 (Life Technologies), essentially as described by others (Gabriel et al., 2001). HVS1 and HVS2 sequences were aligned to the revised Cambridge Reference Sequence (rCRS), (Andrews et al., 1999) and differences scored accordingly. To infer mtDNA haplogroups, mtDNA variants were used to search and build 11 of the mtDNA phylogenetic trees available from http://PhyloTree.org (van Oven and Kayser, 2009).

#### Y-chromosomal genotyping

Thirty-two SNPs at informative positions in the Y-chromosome phylogenetic tree (Karafet et al., 2008) were genotyped using a single-base primer extension (SNaPshot) assay to determine the major Y haplogroups. Twenty-six Y-SNPs were typed in a multiplex single-base primer extension method (SNaPshot, Life Technologies). An additional six Y-SNPs were typed in monoplex reactions using the same method to characterize some relevant sub-haplogroups (Supporting Information Table S2). Detailed methods are explained in Corach et al. (2010). Primer sequences are presented in Supporting Information Table S2. The PowerY kit (Promega) was used according to the manufacturer's description to type 10 Y-chromosomal microsatellite markers.

#### Autosomal genotyping

The Identifiler kit (Life Technologies) was used according to the manufacturer's description to type 17 autosomal microsatellite markers and the Amelogenin locus that was used to confirm the reported gender of each participant.

### Statistical analyses

**Fig. 2:**
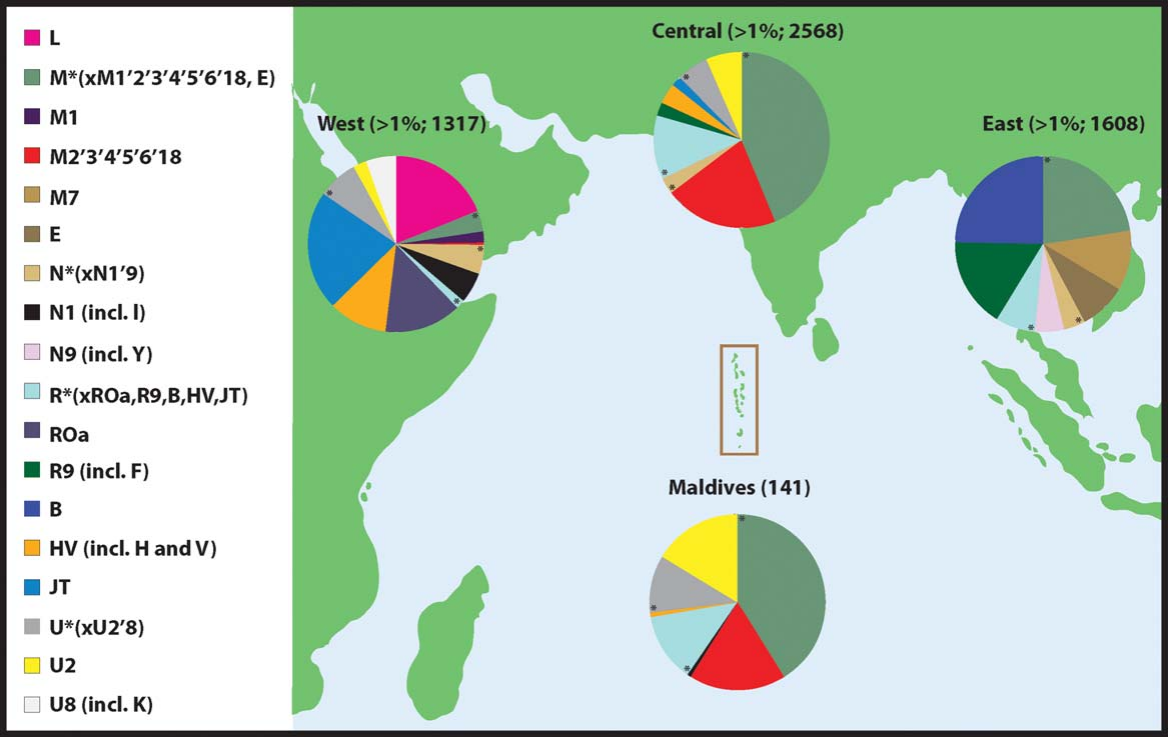
The distribution of mtDNA haplogroup frequencies (>1%) in the Maldives and in the West, Central, and East regional reference populations across the northern Indian Ocean. Complete haplogroup information for the Maldives sample can be found in Supporting Information.

**Fig. 3:**
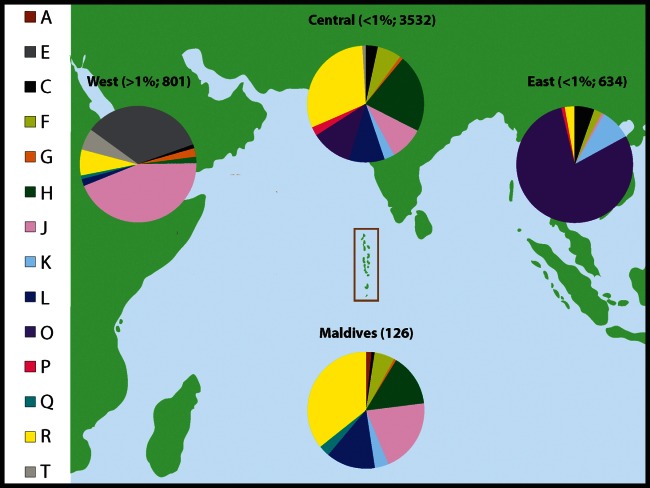
The distribution of Y-haplogroup frequencies (>1%) in the Maldives and in the West, Central, and East regional reference populations across the northern Indian Ocean. Complete haplogroup information for the Maldives sample can be found in Supporting Information Figure S3. The list of haplogroup frequencies in the studies used for the regional reference populations can be found in Supporting Information Table S5.

To assess the most likely parental populations on a large geographic scale, we performed a qualitative comparison of the mtDNA- and Y-haplogroup frequencies found in the Maldives with those found in present-day regions across the Indian Ocean. We constructed three distinct parental reference populations from the literature to represent three coastal geographic regions across the northern Indian Ocean: West, Central, and East (Figs. 2 and 3; see Supporting Information Tables S5 and S6 for details and references). The inclusion of the information in published studies depended on the following criteria for phylogenetic data. (1) We required sufficient phylogenetic detail available for our purpose to discriminate between the three reference populations. Only recent studies were found suitable. (2) We required the phylogenetic resolution across the reference populations to be compatible. Sub-haplogroups were concatenated when needed for comparisons. (3) The haplogroup-specific markers that were typed were required to be used in build 11 of the mtDNA haplogroup phylogeny http://PhyloTree.org (van Oven and Kayser, 2009) or the Y-chromosome (Karafet et al., 2008). Haplogroup frequency distribution pie charts of the Maldives and reference populations were constructed in Excel (Microsoft). Maximum likelihoods estimates for quantitative admixture (Table 2) were obtained using LEADMIX software (Wang, 2003), with the minimum allowed drift set to 1 × 10^−4^, and 10 initial points and 200 integration points for the likelihood calculations.

**TABLE 2:** Maximum likelihood estimates (± 95% CI) of admixture contributions (Wang, 2003) from each parental reference population to the Maldives, for mtDNA-, Y-chromosome, and combined mtDNA and Y haplogroup frequencies

	Reference population		
	
	West^a^	Central^b^	East^c^
mtDNA	0.004 (0.076–<0.001)	0.992 (0.906–0.995)	0.003 (<0.001–0.006)
Y-Chromosome	0.253 (0.194–0.253)	0.746 (0.641–0.746)	<0.001 (0.063–<0.001)
Combined	0.286 (0.285–0.286)	0.713 (0.713–0.713)	<0.001 (0.002–<0.001)

a Saudi Arabia, Qatar, Yemen (including Socotra), Oman, UAE and Somalia.

b India, Pakistan and Sri Lanka.

c Cambodia, Thailand, Malaysia, Indonesia (West), Vietnam (South) and the Philippines.

For a full list of reference population data and literature references, see table S4 (mtDNA) and S5 (Y-chromosome).

**Fig. 4:**
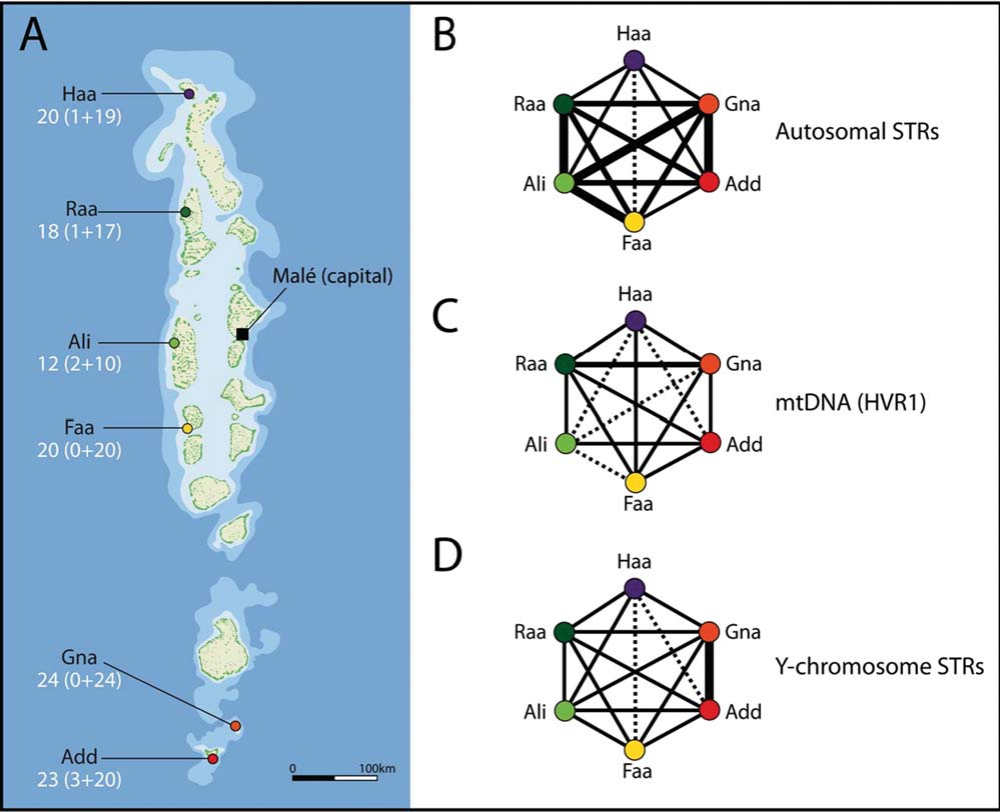
Gene flow between the six main sampling locations measured as 1/Fst (log-transformed and standardized; see Methods for details) for Autosomal STR (4B), mtDNA HVS1 (4C), and Y-STR (4D) data. Solid lines indicate that the two connected locations are not significantly differentiated, with increasing widths indicating higher gene flow. Dashed lines indicate significant differentiation (*P*-value < 0.01). Sampling locations are indicated on the map of the Maldives. The atoll names have been abbreviated using the first three letters of the name, and sample sizes (females + males) are indicated in parentheses (4A).

We estimated genetic diversity, genetic distance, and genetic structure among the six large settlements in the Maldives that were sampled. For autosomal STRs and Y STRs, genetic distances were estimated using Rst under the assumption of a stepwise mutation model (Slatkin, 1995). For mtDNA HVS1 sequences, genetic distances were estimated using the Kimura 2P model for molecular distance and a gamma of 0.25 (Kimura, 1980). To estimate genetic substructure by geography and dialect, we performed an Analysis of Molecular Variance (AMOVA) (Table 3). To estimate genetic diversity and genetic distances, we estimated gene and molecular diversity (Table 4) and pairwise Fst (Fig. 4, Supporting Information Table S3) for autosomal Short Tandem Repeats (STRs), mtDNA HVS1, and Y-chromosome STRs separately using Arlequin (Excoffier et al., 2005). Diversity indices reported are Gene Diversity (expected heterozygosity) for autosomal STRs, haplotype diversity for Y STRs, and sequence diversity (haplotype diversity) and nucleotide diversity for mtDNA HVS1. Pairwise Fst *P*-values were estimated by permutation (>10,000), and corrected for multiple testing using the Holm-Bonferroni procedure (Holm, 1979). For a graphical representation of gene flow between the six main sampling locations (Fig. 4), we transformed pairwise Fst/Rst values: gene flow = log_1.2_(1/Rst). Then, we binned the gene flow values into four classes: (1) significantly low; high Fst, (2) nonsignificant below 20; Fst > 0.025, (3) between 20 and 30; 0.025 > Fst > 0.004, and (4) 30 or higher; Fst < 0.004. No first or second degree familial relatedness was found after pairwise checking for allele mismatch in 17 autosomal STRs. The correlation of genetic distance with geographic distance was assessed using a Mantel test in Alleles In Space (Miller, 2005). Median-joining networks were constructed for mtDNA- (Supporting Information Fig. S1) and Y-STR (Supporting Information Fig. S2A,B) haplotypes using Network 4.6. and Network Publisher 1.3 software (Bandelt et al., 1999). Y-STR markers were given a weight calculated as the inverse of the mutation rate in the dataset to reduce network reticulations. The mtDNA network was redrawn manually for increased graphical resolution and to include haplogroup labels.

**TABLE 3:** Population specific fixation and mean diversity indices for 110 males from 6 major island samples

	Autosomal-STR	mtDNA HVS1		Y-STR
Atoll name	Gene diversity	Sequence diversity	Nucleotide diversity	Haplotype diversity
Add	0.81 (0.05)	0.92 (0.04)	0.013 (0.008)	0.64 (0.14)
Ali	0.77 (0.09)	0.73 (0.08)	0.012 (0.007)	0.44 (0.21)
Gna	0.79 (0.07)	0.95 (0.02)	0.016 (0.008)	0.61 (0.13)
Haa	0.77 (0.08)	0.88 (0.04)	0.013 (0.007)	0.67 (0.08)
Faa	0.79 (0.08)	0.88 (0.04)	0.012 (0.007)	0.57 (0.18)
Raa	0.79 (0.06)	0.92 (0.04)	0.015 (0.008)	0.60 (0.08)
Maldives	0.80 (0.06)	0.96 (0.04)	0.013 (0.001)	0.64 (0.11)

Standard deviations of the mean diversity across loci are indicated in parentheses; HVS1 is treated as a single locus for gene diversity estimation.

**TABLE 4:** Molecular variance (%) and Fst/Rst results from AMOVA per marker type on 110 males from six major island samples

	Aut STR	mtDNA HVS1	Y STR
% Variance within islands	97.34	91.72	92.63
% Variance among islands	2.66^a^	8.28^a^	7.37^b^

a *P* < 0.0001.

b *P* < 0.001.

## RESULTS

### Population origin and admixture

#### mtDNA lineages

We found a total of 63 different mtDNA haplotypes that could be allocated to 29 mtDNA haplogroups, mostly within the M and R clades. An overview of the >1% major haplogroups can be seen in Figure 2; detailed results per island sample are in Supporting Information Table S1 and Figure S1. We were able to identify mostly monophyletic haplogroups, but 26 samples in nine haplotypes could not be monophylogenetically assigned to a haplogroup within macrohaplogroups M and R. These unresolved haplotypes might represent Maldives-specific variants. In particular, within macrohaplogroup R, we detected two previously unreported haplotypes, with HVS1 motifs 16187T-16241T-16319A-16342C (*n* = 6) and 16086C-16209C-16256T (*n* = 5), respectively. Most of the mtDNA haplotypes observed in the Maldives fell into haplogroups M6, M30, M36, M39, M41, M66, R5, U1, and U2 (together accounting for 67.4% of the individuals). All these haplogroups have previously been detected in the Central reference population, that is, South Asia. Four individuals that clearly belonged to haplogroup M2 (defined by 16223T-16274A-16319A), also carried the haplogroup M2b defining mutations 16320T and 195C but lacked other M2b defining variants such as 152C, 182T, 14766C, 16169.1C, and 16189C, therefore most likely reflecting an intermediate split within the M2b haplogroup; we have thus tentatively assigned these individuals to haplogroup “pre-M2b.” Interestingly, all haplogroup M39b individuals in our sample carried a six bp deletion within HVS2 (affecting pos. 105–110), a mutation which is rare but recurrent, as it has previously only been reported on the background of Native American haplogroup D1 (Baeta et al., 2009).

#### Y-chromosome lineages

We found a total of 66 different Y-STR haplotypes, many of which were unique to a single individual. These could be attributed to 10 major haplogroups: A(M191), C(M130), F*(xG,H,I,J,K), G(M201), H(M69), J(M304), K*((xL,M,NO,P,S,T), L(M20), Q(M242), and R(M207) (Fig. 3; detailed results per island sample are in Supporting Information Figs.S2 and S3). The most prevalent haplogroups are F*(xG,H,I,J,K), H(M69), J(M304), L(M20), and R(M207), that combined correspond to 90% of the individuals. Globally, the highest frequencies of haplogroups F*(xG,H,I,J,K), H(M69) (including H(M69)(xH1(M52)) and H1(M52)) and L(M20) are found in the Central reference population, that is, South Asia, whereas they are rare outside this region. The haplogroup J(M304) Y chromosomes are all in subgroup J2(M172). This haplogroup is found at high frequencies in Southwest Asia, in our Western reference population, and in the Caucasus; in South Asia it is widespread at frequencies below 10%, mainly in caste populations. The haplogroup R(M207) Y chromosomes are in subgroups, R1a1a(M17) and R2a(M124). R1a1a(M17) occurs throughout Eurasia, with the highest frequencies in Eastern Europe, Central Asia, and South Asia. R2a(M124) is found at high frequencies in South Asia only. When haplotypes in the median joining Y-STR network are labeled by Y-haplogroup (Fig. S2B), the K*(xL,M,NO,P,S,T (4%) and F(xG,H,I,J,K) (6%) individuals appear to form a monophyletic cluster, whereas H(M69)(xH1(M52)) (6%) individuals appear to be paraphyletic, with at least two distinct lineages. Four Q1a(MEH2) Y chromosomes are found across the network as well. Some of these paraphyletic lineages could be specific to the Maldives.

#### Parental origin

A quantitative analysis of admixture proportions (Wang, 2003) from mtDNA- and Y-haplogroup distributions from three assumed parental populations to the Maldives (Table 2) confirms that the most likely origin of the Maldivian population is in the Central reference population: South Asia. Using LEADMIX, we estimate that 99.2% (95% CI 90.6–99.5) of the mtDNA genomes observed among present-day Maldivians could derive from the Central reference population, with negligible contributions from West or East reference populations. For the Y-chromosome we see a different pattern. Although the majority of the present day Maldivian Y-haplogroups also appear to be shared with the Central reference population [74.6% (64.1–74.6)], about 25% (19.5–25.3) of the Maldivian Y-chromosomes could be contributed by individuals from the West reference population. This is most likely explained by haplogroup J2(M172), which is abundant in Southwest Asia and reaches a higher frequency in the Maldives compared to South Asia. A search for the Y-STR haplotypes within the J2(M172) lineage in the YHRD database (Willuweit and Roewer, 2007) indicated identical or near-identical Y chromosomes in West Asia, Europe, and South Asia.

### Maldivian diversity and population structure

#### Autosomal microsatellites

The six island populations have gene diversity values (Table 3) that indicate a normal outbreeding population (Jorde et al., 2000). The AMOVA (Table 4) indicates a small but significant structure among the islands: 2.7% of the genetic variance (V) in autosomal microsatellites is explained by among-population variance (*V*_among populations_; *P* < 10^−4^) in the AMOVA analyses. This substructure is not related to a dialect difference between northern and southern islands (*V*_among dialect groups_ = 0.42%; *P* = 0.33). Pairwise population comparisons for autosomal markers reveal low pairwise Fst values that indicate little differentiation between islands (Fig. 4B, Supporting Information Table S3). Only the northernmost population from Huvarafushi, Haa atoll, shows a lower gene flow (Fst around 5%) with the other islands. This population is significantly differentiated from Faa. The highest gene flow is found between Ali and proximate atolls. In addition, gene flow is high between the two proximate southern atolls Gna and Addoo. However, over all subpopulations, there was no significant correlation with geographic distance in a Mantel test (*r* = 0.05; *P* > 0.99).

#### mtDNA

Diversity estimates for the island samples and for the combined sample (Table 3) are somewhat lower than typical mainland values but comparable to other island populations (Hurles et al., 2005; Tofanelli et al., 2009; van Oven et al., 2011). There is a significant amount of substructure in the female gene flow: *V*_among populations_ = 8.3% (*P* < 10^−4^) (Table 4). The pairwise Fst values for mtDNA HVS1 are high, indicating reduced female mediated gene flow among the islands compared to the autosomal markers. However, most populations are not significantly differentiated from the others (Fig. 4C, Supporting Information Table S3). The Ali and Haa populations show significantly reduced gene flow with multiple islands. A neighbor-joining network for mtDNA haplotypes (Supporting Information Fig. S1) did not indicate any significant geographic structure in the maternally inherited haplotypic variation. An AMOVA that included dialect groups did not indicate a difference between northern and southern islands (*V*_among dialect groups_ = 0.16%; *P* = 0.20). Moreover, there was no significant correlation of mtDNA HVS1 variation with geographic distance within the archipelago as a whole (*r* = 0.05; *P* > 0.95).

#### Y-chromosome markers

Y-STR diversity is reduced in all island populations as well as in the Maldives as a whole (Table 3). This is most pronounced on Feridhoo Island, on Alifu atoll, where our sample size is smaller (Table 1). The AMOVA analysis show that 7.4% (*P* < 10^−3^) of the genetic variance in Y-STRs is explained by among-population variance (Table 4): male- mediated gene flow that is slightly higher than the female-mediated gene flow. The pairwise island comparisons reveal amounts of male-mediated gene flow that are similar to those for mtDNA. The relatively isolated position of Haa atoll is consistent with the results for autosomal and HVS1 data, with significantly reduced gene flow with Faa and Addoo atolls. However, there are island-to-island patterns that differ between mtDNA and Y markers (Fig. 4D, Supporting Information Table S3). First, there is high male gene flow between Gna and Addoo. Second, there is no apparent reduction in male gene flow among Ali and the other islands. The many pairwise genetic relations among islands are reflected in the near-random structure of the neighbor-joining network for Y-STR haplotypes (Supporting Information Fig. S2A). An AMOVA for dialect groups did not indicate a difference between northern and southern islands (*V*_among dialect groups_ = 0.16%; *P* = 0.20). In addition, no significant correlation with geographic distance across all islands within the archipelago was found (*r* = 0.11; *P* > 0.99).

## DISCUSSION

### Parental population and admixture

The wide range of haplogroups for both mtDNA and Y present in our Maldivian sample suggest a diverse origin. However, our results from both qualitative and quantitative analyses of the haplogroup distribution of uniparentally inherited markers indicate that the varieties of genomes sampled in the Maldives do not differ significantly from those found in the Central reference populations that contain the countries India, Pakistan, and Sri Lanka. In contrast, the most abundant haplogroups in the East reference population [mtDNA haplogroups B, R9, M7, and E, and Y-haplogroups O(M175), C(M130), and K(M9*)] are almost completely missing in our Maldivian samples.

Female-mediated gene flow from the Western region was also limited. Frequent Western mtDNA haplogroups JT, L, R01, and HV are almost completely absent in our Maldivian sample. Based on the quantitative admixture results, a minor contribution from West Asia to the Maldivian Y-chromosome pool is likely. However, this is caused predominantly by sharing of Y-haplogroup J2(M172), a haplogroup that is more frequent in West Asia compared to South Asia. This haplogroup is present at relatively low frequencies in South Asia, predominantly in upper-caste populations (Sengupta et al., 2006). Y-STR haplotypes that are identical or near-identical to those of the Maldivian J2(M172) Y chromosomes are found in a large part of Eurasia, from Central Europe to India. Thus, our analysis is inconclusive about the possibility that most haplogroup J2(M172) Y-chromosomes in the Maldives originate from South Asia, and not directly from West Asia. A more detailed study into this haplogroup in the Maldives may shed further light on this issue.

A previous simulation study of seafaring across the Indian Ocean suggested that there could be an important role for the Maldives in the settling of Madagascar by people from Southeast Asia (Fitzpatrick and Callaghan, 2008). Most simulated traveling scenarios from east to west, and vice-versa, point to an important stopping point in the Maldives. To address this issue in more detail, we compared haplotypes observed in our Maldives sample with those from studies of Malagasy for individuals that belong to macro-haplogroups shared between the two populations. For Y-chromosome data, we used the data by Tofanelli et al. (2009). We compared 10 Y-STR haplotypes in all individuals in subgroups of Y-haplogroups J(M304), L(M20), and R(M207). The closest matching haplotypes found were between a Maldivian individual from Addoo (Mal_045; Supporting Information Table S6) and the single Y-R1a1(SRY10831) individual from Madagascar (Antanosy MAD20) that differ in two repeat differences (one repeat each in DYS390 and DYS391). All other haplotypes differed in at least four repeat differences. Y-R1a1 is most frequent in South Asia and Eastern Europe, and occurs at low frequency elsewhere in the old world. Most likely this Malagasy individual originates from South Asia. For mtDNA, the single Maldivian individual carrying a haplogroup found outside South Asia is an individual from Gaafu Dhaalua atoll, located outside the six major settlements. The individual carries haplogroup M71, found in low frequencies across Southeast Asia. We screened GenBank (http://www.ncbi.nlm.nih.gov/Genbank/) for individuals with a similar haplotype observed in Madagascar using Mitotool (Fan and Yao, 2011). None were found. In conclusion, our results suggest that the proposed Indian Ocean seafaring route through the Maldives did not include migration to these islands.

The ancestry of mtDNA- and Y haplogroups in present-day South Asia is a highly complex mix of tribal, caste, religious, and language groups, and the structure along those cultural identities is likely to be subtle (Majumder, 2010). This may reduce the potential of an in-depth comparative analysis with the Maldives, but some interesting observations can be made. As Maldivian society has a long history of Islamic influence, a genetic link with South Asian Muslim populations could be expected. However, Eaaswarkhanth et al. (2010) report that Muslims and non-Muslims in India largely have the same Y-haplogroup frequency distribution, except that in Muslims low frequencies of Y-E1b1b1a(M78), Y-J(M304)(xJ2(M172)), and Y-G(M201) are found that are absent in non-Muslims (Eaaswarkhanth et al., 2010). In our Maldivian sample, none of those Y-haplogroups were found. The same study also found mtDNA haplogroup sharing across religious affiliations, with the exception of the presence of a L0 sub-haplogroup in Muslims only; this haplogroup was also not observed in our Maldivian sample. This suggests that Muslim Indians did not contribute more significantly to the Maldivian ancestral population than non-Muslims Indians, and that the Maldivians were converted by cultural diffusion. The conversion of the Maldives to Islam in the twelfth century should, therefore, be seen as independent from the earlier Islamization of the Indian sub-continent.

The Dhivehi language is the southernmost Indo-Aryan language, and sharing of specific haplogroups with Indo-Aryan Sinhalese populations mostly from northern India and from Sri Lanka could point to a common origin of these populations. The Indo-Aryan-speaking higher castes in northern India in particular show moderately high levels of admixture with West Eurasian populations that include Indo-Aryan speakers (Bamshad et al., 2001; Basu et al., 2003; Reich et al., 2009). However, the consensus is that most of the haplogroup diversity in uniparental markers in South Asia originates from an early out-of-Africa migration at least 40K YBP (Kivisild et al., 2003; Karafet et al., 2008), with some association with demic diffusion of agriculture over 8K YBP ago (Kivisild et al., 2003; Basu et al., 2003; Cox et al., 2012), and that there is little relation to language structure. The investigation of the history and distribution of the Indo-Aryan languages remains a challenge and requires a more complete and more detailed view of population structure in Eurasia. The current study provides a reference for future studies into the evolution of the Indo-Aryan languages.

We observed low frequencies of some haplogroups in the Maldives that are typically found outside South Asia. This may indicate that occasional migration from outside South Asia to the Maldives did occur. We found one Maldivian with mtDNA haplogroup M71, which has been described to occur at a low frequency in Southeast Asia (Peng et al., 2010; Tabbada et al., 2010; Kong et al., 2011). In addition, we found two Y-chromosome haplogroup A(M91) carriers on Gnaviyani. This Y-haplogroup occurs at relatively low frequencies throughout Africa, and is assumed to be one of the deepest-rooting lineages of human Y chromosomes (Karafet et al., 2008). It is rarely found outside Africa apart from recent migration events. The two Y chromosomes have an identical Y-STR haplotype; our search with the seven most abundant loci in the YHRD database (Willuweit and Roewer, 2007) revealed identical haplotypes in populations in Ethiopia and Uganda. These results confirm the mentioning of recent migration from East Africa to Gnaviyani. In contrast, the reported African ancestry of the people on Feridhoo Island is not supported by the presence of typical ‘African’ mtDNA and Y-chromosome haplogroups.

### Maldivian population structure

Diversity estimates in autosomal and mtDNA markers reveal normal to mildly reduced diversity levels that are typical of other island populations (Jorde et al., 2000; van Oven et al., 2011). The diversity indices for Feridhoo Island, Ali atoll, are lower than for the other islands. The smaller sample size precludes drawing conclusions about gene flow between Ali and the other atolls. Y-chromosomal diversity is strongly reduced on each island and in the total sample of the Maldives. The values are lower than for many islands and comparable to populations that have experienced a bottleneck or founder effect (van Oven et al., 2011). We observed a pattern of genetic variation in the Maldives that indicates differentiation between some atolls. We find that significant amounts of the genetic variance in all marker systems are explained by among-population structures. In particular, both female-mediated gene flow and male-mediated gene flow are reduced. Male-mediated gene flow is typically reduced in most human populations due to patrilocality (Destro-Bisol et al., 2004; Hamilton et al., 2005; Wood et al., 2005; Chaix et al., 2007). However, the reduced variation in Y-chromosomal makers combined with reduced male-mediated gene flow suggests that all islands experienced independent founder effects. The reduced female-mediated gene flow is usually only found in populations where matrilocality is practiced (Hamilton et al., 2005). Although rare today, it is consistent with the historical mention of matrilocality in the Maldives (Metcalf, 2009). From pairwise population comparisons of uniparental marker diversity, some interesting patterns emerge. Haa atoll seems to be the most differentiated island population, perhaps not surprising given its remote location within the archipelago. Significant female-mediated gene flow between Raa and Gnaviyani suggests a historical maternal link between these atolls. Pairwise Rst values from Y-STR data reveal higher gene flow between Addoo and Gnaviyani, as expected from their relative geographic proximity and their sharing of the local southern dialect. However, a clear division between northern- and southern-dialect speakers is not apparent in the Y-chromosome or in the mtDNA data. Similarly, despite the observed reduced gene flow for both uniparental markers among some of the island populations, an obvious geographic structure is not apparent from the haplotype variation.

## CONCLUSION

We report a strong genetic link between the Maldives Islands and the Indian sub-continent. We exclude the sharing of haplogroups between Southeast Asia and the Maldives, which questions the previously suggested central role of a Maldive Islands' stopping point for migration from South East Asia to Madagascar. The Maldives were more likely used for supplies than for settling a population, which is no surprise considering the limited landmass of coral atolls. We also dismiss female-mediated gene flow from West Asia. Male-mediated gene flow from West Asia to the Maldives could have occurred, although it cannot be separated from such gene flow through South Asia. The wide range of Y-chromosomal and mtDNA haplogroups mirrors the haplogroup diversity in South Asia. We find a subtle substructure within the Maldives that is not directly related to geographic distance or dialect. Reduced diversity of Y-chromosomal markers on each atoll combined with reduced male-mediated gene flow between atolls suggests independent founder effects for each atoll. Reduced female-mediated gene flow between atolls confirms a Maldives-specific history of matrilocality.
