# Supplementary figures and images for: Indian Ocean Crossroads: Human Genetic Origin and Population Structure in the Maldives
Source: Am J Phys Anthropol. 2013 Mar 21;151(1):58–67. doi: 10.1002/ajpa.22256 (PMC3652038; doi:10.1002/ajpa.22256)

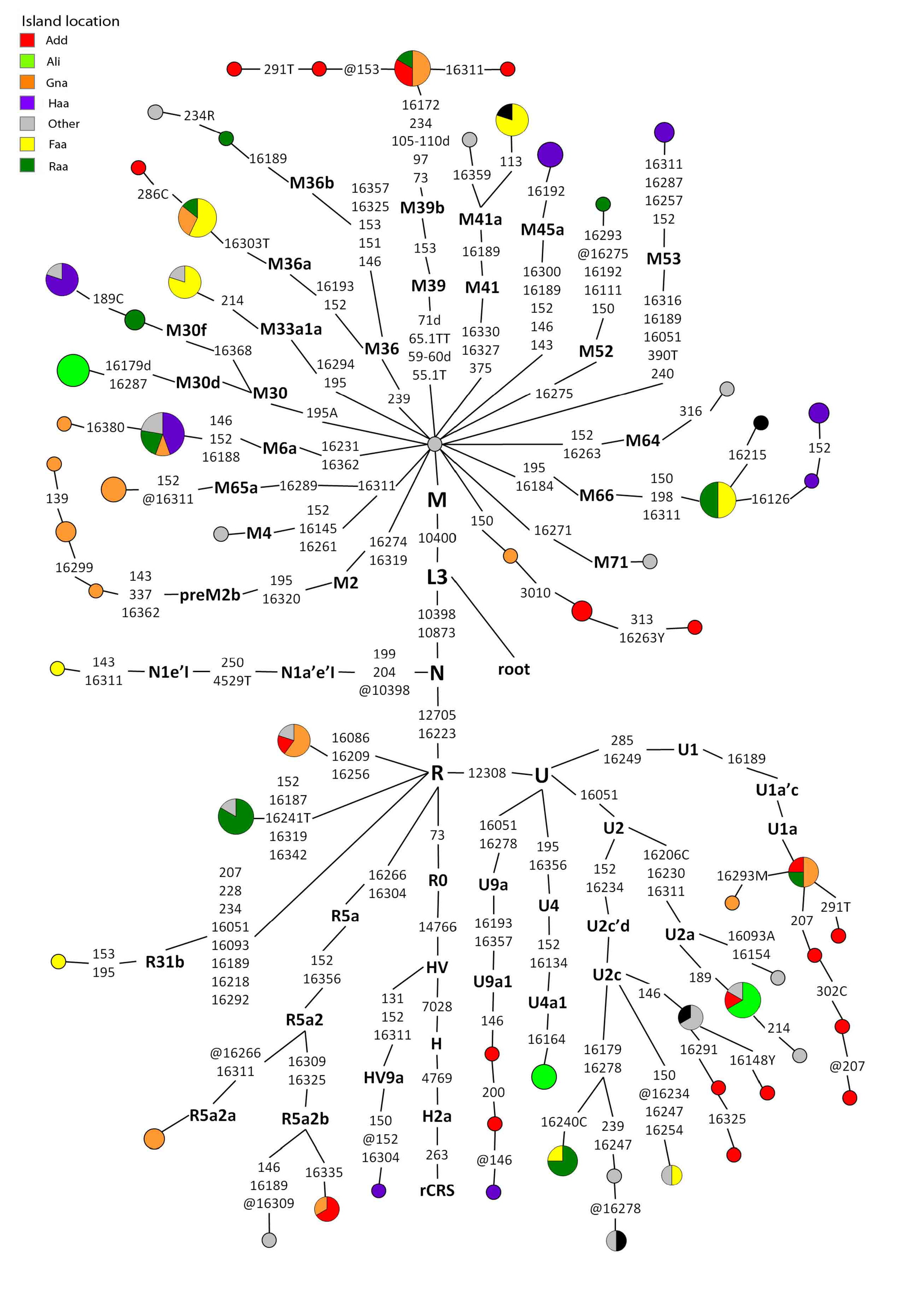

Supplement: Supplementary file 1 [file ajpa0151-0058-SD1.tif]

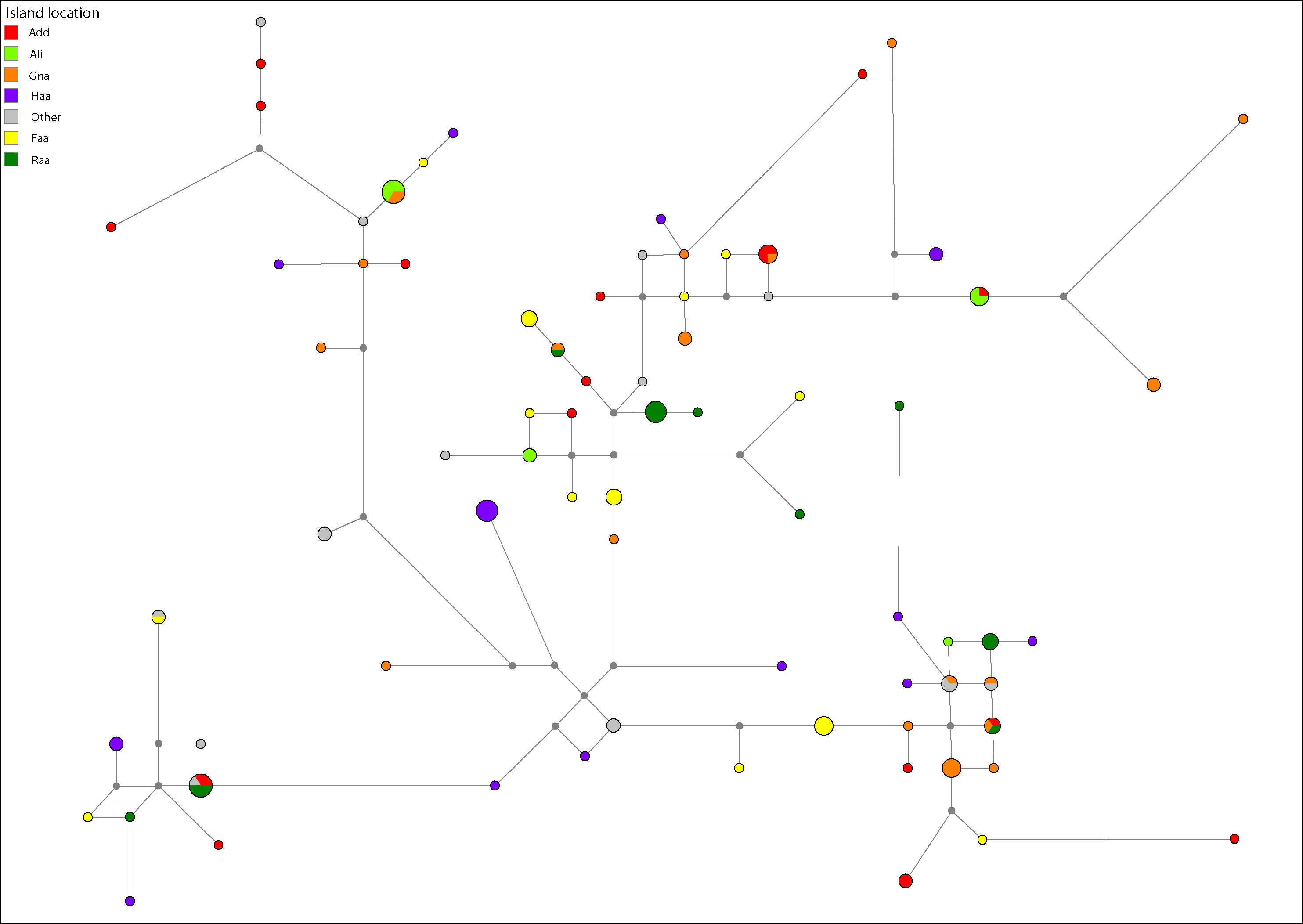

Supplement: Supplementary file 2 [file ajpa0151-0058-SD2.tiff]

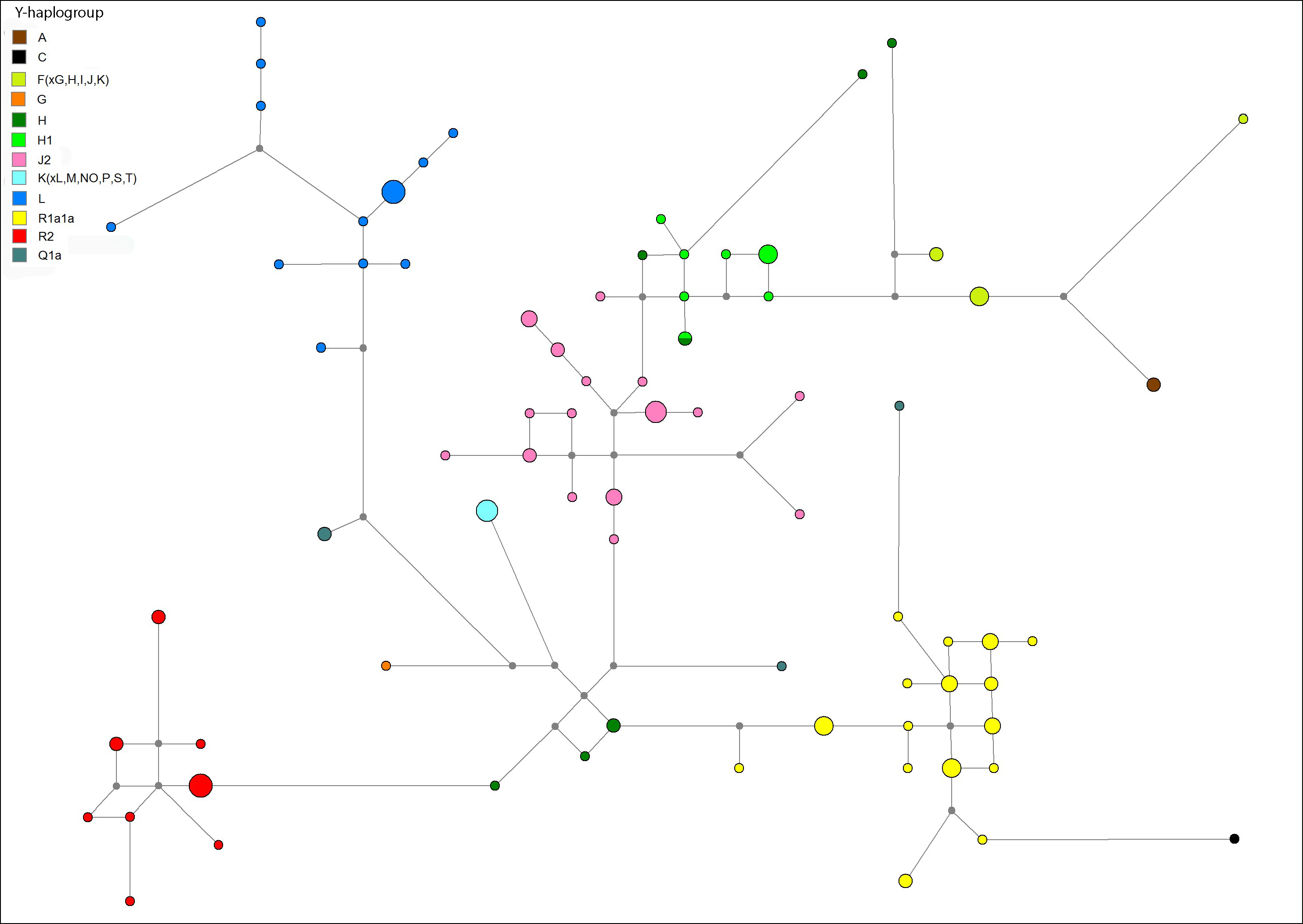

Supplement: Supplementary file 3 [file ajpa0151-0058-SD3.tiff]
