# Supplementary material for: Indian Ocean Crossroads: Human Genetic Origin and Population Structure in the Maldives
Source: Am J Phys Anthropol. 2013 Mar 21;151(1):58–67. doi: 10.1002/ajpa.22256 (PMC3652038; doi:10.1002/ajpa.22256)
Supplement: Supplementary file 5 [file ajpa0151-0058-SD5.doc]

mtDNA Haplogroup	Add	Ali	Gna	Haa	Faa	Raa	Other	Maldives total	
HV9a				1				1	
M	3		1				1	5	
M30d		5						5	
M30f				5		2		7	
M33a1a				1	4			5	
M36a	1		2		4	1		8	
M36b						1	1	2	
M39b	5		3			1		9	
M4*							1	1	
M41a					4		2	6	
M45a				3				3	
M52						1		1	
M53				2				2	
M64							1	1	
M65a**			3					3	
M66***				3	3	3	1	10	
M6a			2	6		2		10	
M71							1	1	
N1e'I					1			1	
pre‐M2b			4					4	
R	1		3	1		5	1	11	
R31b					1			1	
R5a2a			2					2	
R5a2b	2		1		1			4	
U1a	5		3			1		9	
U2a	1	4					3	8	
U2c	3			1	2	3	6	15	
U4a1		3						3	
U9a1	2			1				3	
Total	23	12	24	24	20	20	18	141	

* formerly M4a									
** formerly M4b1									
*** formerly M4c									

Nomenclature from PhyloTree (Build 11); data from HVS1 and 2 sequence, and 17 coding region SNPs
