# Supplementary material for: Indian Ocean Crossroads: Human Genetic Origin and Population Structure in the Maldives
Source: Am J Phys Anthropol. 2013 Mar 21;151(1):58–67. doi: 10.1002/ajpa.22256 (PMC3652038; doi:10.1002/ajpa.22256)
Supplement: Supplementary file 6 [file ajpa0151-0058-SD6.doc]

Locus	dbSNP
accession	
26
Haplogroup	
Forward Primer	
Reverse Primer	Amplicon
Size (bp)	
Minisequencing Primer	
Orientation	Variants**
(wildtype/
mutant)	
M9	rs3900	K	ctgcaaagaaacggccta	gaacgtttgaacatgtctaaa	70	ACTGCAAAGAAACGGCCTAAGATGGTTGAAT	Forward	C/G	
M20	rs3911	L	agttggccctttgtgtctgt	catgttcagtgcaaatgcaa	106	cgtgaaagtctgacaaCACATTTGTAGGTTCAACCAACTGTGGATTGAAAAT	Forward	A/G	
M45	rs2032631	P	gagagaggatatcaaaaa	tgacagtggcaccaaaggt	138	aacaaCTCAGAAGGAGCTTTTTGC	Reverse	C/T	
M69	rs2032673	H	tgggtagcctgttcaaatcc	tctccccttagctctcctgtt	117	tgccacgtcgtgaaagtctgacaaGGCTGTTTACACTCCTGAAA	Forward	T/C	
M70	rs2032672	T	aggcaccatctgtgaaaac	ctgagggctggactataggg	115	cgtcgtgaaagtctgacaaATTCTGTTGTGGTAGTCTTAG	Reverse	T/G	
M96	rs9306841	E	gccagccaagaatgaaga	tgagctgtgatgtgtaacttgg	143	GGAAAACAGGTCTCTCATAATA	Reverse	G/C	
M124	-	R2	agcaaagttgaggttgcac	tgggcaacaccagaatcta	93	CCCCCCCCCCCCCCaggtgccacgtcgtgaaagtctgacaaGGGGAACAGGGA	Reverse	C/T	
M145	rs3848982	DE	cctcccactcctttttggat	gcatacttgcctccacgact	96	gactaaactaggtgccacgtcgtgaaagtctgacaaTAGACACCAGAAAGAAAGGC	Reverse	G/A	
M170	rs2032597	I	ttatgttttcatattctgtgcatt	tgagacacaacccacactg	90	CAACCCACACTGAAAAAAA	Reverse	T/G	
M173	rs2032624	R1	ttttcttacaattcaagggcat	ctgaaaacaaaacactggc	81	CaactgactaaactaggtgccacgtcgtgaaagtctgacaaTACAATTCAAGGGCATTT	Forward	T/G	
M174*	rs2032602	D	cgcttctctgaataccttctgg	gacccatcttgcaaggaaa	101	gtcgtgaaagtctgacaaCCTTCTGGAGTGCCC	Forward	TT/TC*	
M175	rs2032678	O	gatttaaactctctgaatcag	ttctactgatacctttgtttctgtt	79	acgtcgtgaaagtctgacaaCACATGCCTTCTCACTTCTC	Forward	T/A	
M181	rs2032599	B	caaagttggcttgggatttt	tggcaatatttactatttggca	105	CCCCCCaactgactaaactaggtgccacgtcgtgaaagtctgacaaCAAGCTACTACCT	Reverse	A/G	
M201	rs2032636	G	gatctaataatccagtatca	ccagcatcctatcagcttca	75	CaactgactaaactaggtgccacgtcgtgaaagtctgacaaCTAAGTACCTATTACGAA	Reverse	C/A	
M207	rs2032658	R	ggggcaaatgtaagtcaag	tcacttcaacctcttgttggaa	83	tgacaaAAGGTATTGTTATTCTCTTT	Reverse	C/T	
M213	rs2032665	F	ggccatataaaaacgcag	aaaatattcagaacttaaaa	131	acaaTCAGAACTTAAAACATCTCGTTAC	Reverse	A/G	
M214	rs2032674	NO	ggaaagaaaaagaatgct	agcctgggagacagtgtga	95	actaaactaggtgccacgtcgtgaaagtctgacaaAGACACTGTCTGAAAACAAC	Reverse	A/G	
M231	rs9341278	N	tggaaaatgtgggctcgt	tgacgatctttcccccaat	120	ctgactaaactaggtgccacgtcgtgaaagtctgacaaTTTACTGTTTCTACTGCTTTC	Forward	G/A	
M242	rs8179021	Q	gcaaaaaggtgaccaagg	gggctttcagcataatacctt	140	acgtcgtgaaagtctgacaACGTTAAGACCAATGCCAA	Reverse	G/A	
M304	rs1344735	J	aggcaaagaaaagcagg	aaacgtcttataccaaaatat	113	ggtgccacgtcgtgaaagtctgacaaTTGAAAGTAACTTGTGA	Forward	A/C	
M343	rs9786184	R1b	cgtagcccgagagaaaac	catagccacccccacatatc	99	ctaaactaggtgccacgtcgtgaaagtctgacaaTGCCCTCGTGTTCCA	Forward	C/A	
M91	rs2032651	A	tccccctacattgctattctg	cttaccttttgcgtatttttcaa	96	gtctgacaaTTGCTATTCTGTTTTTTTT	Forward	T/A	
P143	rs4141886	C-F	cccaacttcggtttttggta	tcatccaaggagcaacaca	85	ctaggtgccacgtcgtgaaagtctgacaaGAAAATGTGTGGGGTT	Forward	G/A	
P202	-	S	tttttaaacttcccagtttgtgg	tgtgtcacaaaaccatctgct	89	tcgtgaaagtctgacaaTGTTACTTTTCTCAGGCTTAT	Reverse	A/T	
P256	-	M	tcttggttttcccattgacc	cccaacttgtctgtgccttc	86	CCCaactgactaaactaggtgccacgtcgtgaaagtctgacaaCCTACACTAGATAGAA	Forward	G/A	
SRY10831***	rs2534636	B-R/R1a	ccacataggtgaaccttga	tcatccagtccttagcaacca	150	TCTGGCCTCTTGTATCTGACTTTTTCACACAGT	Reverse	A/G/A	
SRY4064	rs9786608	E	ggtatgacaggggatgatg	ccacgcccagctaattttttgt	225	aggtgccacgtcgtgaaagtctgacaaGTAATCCCAGCCCTTCGAGAGGTCAAG	Forward	G/A	
M130	rs3528497	C	tatctcctcttctattgcag	ccacaagggggaaaaaac	205	CCCCCCCCCCCCCCCCCCCCCaactgactaaactaggtgccacgtcgtgaaagtctg	Forward	C/T	
M52	-	H1	cctcaacttcccagagtgttg	gacgaagcaaacatttcaa	152	cgtcgtgaaagtctgacaaAATATCAAGAAACCTATCAAACATCC	Reverse	T/G	
M189	rs2032606	M1	ttcttaggctttggggtgaa	ttacctatgtgggcagcctt	185	cgtcgtgaaagtctgacaaTTTAGATGAAGCTTTAAAA	Forward	G/T	
MEH2*	rs4252209	Q1a	tttgagtaagccatcacccc	tgcaaaaactgcattgatga	83	CCCCCCCCCaactgactaaactaggtgccacgtcgtgaaagtctgacaaATGTAATTTA	Forward	GG/GT*	
M17	rs3908	R1a1a	cctggtcataacactggaa	agctgaccacaaactgatgt	171	gtcgtgaaagtctgacaaCCAAAATTCACTTAAAAAAACCC	Reverse	G/C	
									
* The PCR of MEH2 and M174 also amplify a X-chromosomal fragment. Because of this fragment there will always be 2 peaks for samples with the derived allele	
** Variants are displayed as how they appear in the SNaPshot-profile	
*** SRY10831 is a recurrent mutation, hg B-R carry the derived allele and hg A and R1a carry the original allele	
