# Supplementary material for: Indian Ocean Crossroads: Human Genetic Origin and Population Structure in the Maldives
Source: Am J Phys Anthropol. 2013 Mar 21;151(1):58–67. doi: 10.1002/ajpa.22256 (PMC3652038; doi:10.1002/ajpa.22256)
Supplement: Supplementary file 7 [file ajpa0151-0058-SD7.doc]

Autosomal‐STR

Island	Add	Ali	Gna	Haa	Faa	Raa	
Add		0.141±0.008	0.37402±0.016	0.010±0.003	0.010±0.003	0.116±0.009	
Ali	0.024		0.340±0.013	0.063±0.008	0.567±0.014	0.582±0.014	
Gna	0.001	0.004		0.020±0.004	0.107±0.010	0.257±0.012	
Haa	0.055	0.047	0.044		0.003±0.001	0.014±0.004	
Faa	0.038	‐0.007	0.011	0.071		0.191±0.009	
Raa	0.017	‐0.007	0.005	0.057	0.009		


mtDNA HVS1

Island	Add	Ali	Gna	Haa	Faa	Raa	
Add		0.014±0.004	0.028±0.005	<0.001	0.008±0.003	0.048±0.007	
Ali	0.11		0.002±0.001	<0.001	<0.001	0.004±0.002	
Gna	0.048	0.15		<0.001	0.005±0.003	0.13±0.010	
Haa	0.12	0.18	0.075		<0.001	0.039±0.005	
Faa	0.075	0.19	0.073	0.067		0.031±0.005	
Raa	0.047	0.13	0.023	0.051	0.058		


Y-STR

Island	Add	Ali	Gna	Haa	Faa	Raa	
Add		0.183±0.012	0.886±0.011	0.0020±0.001	0.061±0.009	0.065±0.007	
Ali	0.030		0.149±0.010	0.022±0.005	0.030±0.005	0.020±0.004	
Gna	‐0.029	0.047		0.015±0.003	0.059±0.008	0.081±0.010	
Haa	0.139	0.156	0.093		0.001±0.000	0.014±0.003	
Faa	0.048	0.120	0.054	0.159		0.139±0.009	
Raa	0.055	0.167	0.047	0.100	0.035		
