# Supplementary material for: Indian Ocean Crossroads: Human Genetic Origin and Population Structure in the Maldives
Source: Am J Phys Anthropol. 2013 Mar 21;151(1):58–67. doi: 10.1002/ajpa.22256 (PMC3652038; doi:10.1002/ajpa.22256)
Supplement: Supplementary file 8 [file ajpa0151-0058-SD8.doc]

Region	Country	Reference


West	Oman	Rowold et al. 2007	18				2	3	4		3	17		16	17		3	7	6	9	105	
West	Qatar	Rowold et al. 2007	14	3				6	4		3	7		11	21		1	8	9	3	90	
West	Saudi Arabia	Abu-Amero et al. 2008	54	19		3		16	44		22	98		64	150		1	52	4	25	552	
West	Somalia	Cerny et al. 2009	27																		27	
West	United Arab Emirates	Rowold et al. 2007	12					10	4		20	8	1	23	18		3	8	11	13	131	
West	Yemen	Cerny et al. 2008	61	2		3		2	5		3	24		14	49		8	5		9	185	
West	Yemen	Kivisild et al. 2004	54	1				8	9		2	4		7	8		2	11		9	115	
West	Yemen	Rowold et al. 2007	6	3				4	2			4		4	12		1	6	4	4	50	
West	Yemen (Socotra)	Watson et al. 1996, 1997	3						4		16	25		2	14		1				65	
Central	India	Cordeaux et al. 2003*			2	135		376	2		37	1	8	11	11	42	47	58	20	2	752	
Central	India	Kivisild et al. 1999*	1			54		99	5		13	1		14	7	7	46	10	32		289	
Central	India	Kivisild et al. 2003*				60		86			2						26		1		175	
Central	India	Metspalu et al. 2004				135		344	4		23	2	4	43	21	14	90	50	60	1	791	
Central	India	Thanseem et al. 2006				155		77			1			3	8		66	8	27	2	347	
Central	India	Watkins et al. 2008						92			1			10	1		16	18	17		155	
Central	Pakistan	Quintana-Murci et al. 2004	1					49	3		2	2		16	2		8	6	11		100	
East	Borneo	Hill et al. 2007**			16		22	40		4	6		38			24	7				157	
East	Java	Hill et al. 2007**			6		1	11		2	7		5			13	1				46	
East	Malaysia	Haslindawaty et al. 2010			23	10	21	58		11	7		64			34	17	3			248	
East	Malaysia	Maruyama et al. 2010	1		13	1	12	38		3	3		26			22	3		1	1	124	
East	Malaysia	Hill et al. 2006 (n=260) and Fucharoen et al. 2001 (n=20)**			9			98		17	28		22			52	54				280	
East	Philippines	Tabbada et al. 2010			74		69	34		20	2		140			65	19				423	
East	Sumatra	Hill et al. 2006**			25		12	42		18	7		42			30	3	1			180	
     East 	Vietnam 	Peng et al. 2010 	13 	2 	39 	12 	3 	62 	26 	11 	168 	
total	252	28	181	556	141	1532	90	87	211	193	412	238	339	329	434	251	203	78	5555

*data extracted from: Metspalu et al. 2004
**data extracted from: Peng et al. 2010
