# Supplementary material for: Indian Ocean Crossroads: Human Genetic Origin and Population Structure in the Maldives
Source: Am J Phys Anthropol. 2013 Mar 21;151(1):58–67. doi: 10.1002/ajpa.22256 (PMC3652038; doi:10.1002/ajpa.22256)
Supplement: Supplementary file 9 [file ajpa0151-0058-SD9.doc]

Region	Country	Reference	A	B	DE	D	E		C	F	G	H	I	J	K(xL,M,N)	L	M	NO	N	O	P	Q	R	S	T	total	
West	Oman	Giacomo et al. 2004												5												5	
West	Oman	Cruciani et al. 2004					2	 																		2	
West	Oman	Luis et al. 2004	1				28		4		2	2		58		1		1			1		13		10	121	
West	Qatar	Cadenas et al 2008*		2			10				2	1		48		2							7			72	
West	Saudi Arabia	Abu-Amero et al 2009		3			26		2		5	3		91		3				1		4	11		8	157	
West	Saudi Arabia	Karafet et al. 2005			2						1			8		5						1	5			22	
West	Somalia	Sanchez et al 20058	1	2			167				1	1		6									2		21	201	
West	United Arab Emirates	Cadenas et al 2008*					28		2	7	7	7		74		5				2		3	20		8	163	
West	United Arab Emirates	Giacomo et al. 2004												13												13	
West	United Arab Emirates	Cruciani et al. 2004					6																			6	
West	Yemen	Cadenas et al 2008*					10				1			51												62	
Central	India	Spencer Wells et al. 2001							13	32		45		28		77				4	5		56			260	
Central	India	Kivisild et al. 2003							17	18	1	65		38	1	38				1	2		143		1	325	
Central	India	Cordaux et al. 2004			2				29	57		71		16	30	24				24	55		40			348	
Central	India	Karafet et al. 2005							10	40	6	110		35		49				40	3	1	111			405	
Central	India	Sengupta et al. 2006							13	38	9	192		68		49				167		3	189			728	
Central	India	Sahoo et al. 2006				4			10	23		169		49	61	39				127	20	1	317		32	852	
Central	India	Thanseem et al. 2006							9	12		64		37	7	22				26			73			250	
Central	Pakistan	Underhill et al. 2000							1	1		3		4	2	3				1	2		21			38	
Central	Pakistan	HGDP@FLDO 2005					3		13	1	11	11	1	26		23				4		6	77			176	
Central	Pakistan + India	Underhill et al. 2000		2			3		1	7		6		13	2	10				2	2		39		1	88	
Central	Sri Lanka	Karafet et al. 2005							3	9	5	23		18		14				1		3	15			91	
East	Cambodia	Wise et al. 2005													3					16	1		1			21	
East	Cambodia	Shi et al. 2005																		2						2	
East	Cambodia	HGDP@FLDO 2005										1							1	4						6	
East	Cambodia + Laos	Underhill et al. 2000				1			1			1		2	1					11			1			18	
East	Indonesia	Karafet et al. 2005							1	1					1					16			2			21	
East	Indonesia	Hurles et al. 2005							5					1	1					14			1			22	
East	Indonesia	Kayser et al. 2006**			1				6	11					7			2		112	4		7	2		152	
East	Malaysia	Karafet et al. 2005			1				1		2				2		1			24			1			32	
East	Malaysia	Hurles et al. 2005							10	1				1					1	49			3			65	
East	Malaysia	Wise et al. 2005													1					11						12	
East	Malaysia	Kayser et al. 2006**							2	1					2					12			1			18	
East	Philippines	Han-Jun Jin et al. 2003							3						8					63						74	
East	Philippines	Karafet et al. 2005							1						23					24						48	
East	Philippines	Hurles et al. 2005													1					27						28	
East	Philippines	Kayser et al. 2006**							4	1					1					32			1			39	
East	Thailand	Han-Jun Jin et al. 2003			1										3					47						51	
East	Thailand	Wise et al. 2005													1					17	1					19	
   East 	Thailand 	Shi et al. 2005 	21 	21 	
Total	2	9	7	5	283	161	260	53	775	1	690	158	364	1	3	2	902	96	22	1157	2	81	5034
*data extracted from: Abu-Amero et al 2009
**See also Kayser et al. 2003
